# Elucidation of Anti-Obesity Mechanisms of Phenolics in *Artemisiae argyi* Folium (Aiye) by Integrating LC-MS, Network Pharmacology, and Molecular Docking

**DOI:** 10.3390/life14060656

**Published:** 2024-05-22

**Authors:** Yongxiang Liu, Jinhai Luo, Baojun Xu

**Affiliations:** 1 Guangdong Provincial Key Laboratory IRADS and Department of Life Sciences, BNU-HKBU United International College, Zhuhai 519087, China; liuyongxiang@uic.edu.cn (Y.L.); luojinhai@uic.edu.cn (J.L.); 2 School of Chinese Medicine, Hong Kong Baptist University, Hong Kong, China

**Keywords:** *Artemisiae argyi* Folium, obesity, LC-MS, network pharmacology, molecular docking

## Abstract

The global prevalence of obesity is a pressing health issue, increasing the medical burden and posing significant health risks to humans. The side effects and complications associated with conventional medication and surgery have spurred the search for anti-obesity drugs from plant resources. Previous studies have suggested that *Artemisiae argyi* Folium (Aiye) water extracts could inhibit pancreatic lipase activities, control body weight increase, and improve the plasma lipids profile. However, the exact components and mechanisms were not precisely understood. Therefore, this research aims to identify the chemical profile of Aiye and provide a comprehensive prediction of its anti-obesity mechanisms. The water extract of Aiye was subjected to LC-MS analysis, which identified 30 phenolics. The anti-obesity mechanisms of these phenolics were then predicted, employing network pharmacology and molecular docking. Among the 30 phenolics, 21 passed the drug-likeness screening and exhibited 486 anti-obesity targets. The enrichment analysis revealed that these phenolics may combat obesity through PI3K-Akt signaling and MAPK, prolactin, and cAMP signaling pathways. Eight phenolics and seven central targets were selected for molecular docking, and 45 out of 56 docking had a binding affinity of less than −5 kcal/mol. This research has indicated the potential therapy targets and signaling pathways of Aiye in combating obesity.

## 1. Introduction

Obesity is caused by the pathological excessive deposit of body fat [1]. A body mass index (BMI) higher than 30 kg/m^2^ indicates the clinical sign of adult obesity [2]. Nowadays, obesity has almost become a global epidemic, except in some African and Asian countries [3]. The 2022 data from the Centers for Disease Control and Prevention (CDC) reported that obese adults accounted for no less than 35% of the 22 states of the United States [4]. The global prevalence of overweight and obesity for both boys and girls will exceed 25% by 2030 [5]. Obesity is positively correlated with various diseases, including diabetes mellitus (type II), high blood pressure, cardiovascular diseases, certain kinds of cancer, osteoarthritis, fatty liver, gallbladder diseases, sleep apnea, etc. [3,6,7,8]. The epidemic of obesity slows down economic development while increasing medical costs worldwide. Meanwhile, obesity decreases work efficiency and reduces the quality of life of the people suffering from it [9]. The mechanisms of anti-obesity drugs include diminishing the appetite, inhibiting the absorption of fat, and increasing energy expenditure [2]. The side effects of those anti-obesity drugs include diarrhea, steatorrhea, malnutrition, pancreatitis, gastroparesis, cholelithiasis, nephrolithiasis, severe hypertension, anxiety, depression, cognitive impairment, suicidal tendency and behavior, and congenital disabilities [2,7,10,11]. Weight-loss surgery is carried out when medication interventions are not effective in the weight control of severely obese patients, and the complications of weight-loss surgery include postoperative mortality, wound infection, ulcers, intestinal obstruction, deficiency of micronutrients, etc. [8]. Due to the commonly existing side effects or complications of medications or surgery, the development of novel anti-obesity drugs from natural plant sources, which are safer and more effective, becomes a priority.

*Artemisiae argyi* Folium (Aiye) has been used for thousands of years as an herbal medicine to treat gynecological, skin, digestive, and respiratory diseases. Pharmacological research has shown that it has anti-virus, anti-bacteria, anti-oxidative, anti-inflammatory, anti-dermatitis, gastro-protective, and anti-cancer effects, mainly due to the presence of essential oils, flavonoids, and organic acids [12,13,14,15,16,17,18]. Pharmacological research has identified various anti-cancer components of Aiye, including jaceosidin, irigenin, luteolin, eriodictyol, apigenin, eupatilin, casticin, and artemetin [19,20,21,22,23,24,25,26,27,28,29]. In obesity, the secretion of adipokines by adipose tissue was dysregulated, characterized by the up-regulation of proinflammation adipokines and the down-regulation of anti-inflammation adipokines [30]. Furthermore, the prolactin signaling pathway participated in adipogenesis and energy balance [31]. The MAPK pathway is involved in appetite regulation, thermogenesis, adipogenesis, and glucose homeostasis, which is tightly connected with obesity [10]. The PI3K-Akt pathway participated in the proliferation and growth of cells, while the cAMP signaling pathway involved insulin secretion [10]. All those pathways are promising for the treatment of obesity. The anti-inflammation and anti-oxidation effects of Aiye’s phenolic acids and flavonoids indicated that Aiye may combat the progress of obesity. A variety of bioactive components of Aiye possess anti-oxidation and anti-inflammation effects, including 7-hydroxycoumarin, daphnetin, nepetin, 5,6,4′-trihydroxy-7,3′-dimethoxyflavone, apigenin, kaempferol, hispidulin, jaceosidin, irigenin, and cirsimaritin [32,33,34,35,36,37,38,39,40,41,42,43,44,45,46,47,48,49,50]. Furthermore, apigenin ameliorates insulin resistance induced by obesity, while cirsimaritin possesses anti-diabetes effects and ameliorates liver disease caused by a high-fat diet [49,51,52]. The scopoletin attenuates the toxicity of high glucose [53,54]. The chlorogenic acid, neochlorogenic acid, isochlorogenic acid A, and isochlorogenic acid B of Aiye inhibit the activities of pancreatic lipase [55]. The administration of water extracts derived from Aiye significantly decreased the body weight of high-fat-diet-fed mice, enhanced their plasma lipid profile, and mitigated glucose intolerance and hepatic steatosis [56]. However, the exact components of Aiye’s water extracts that confer anti-obesity effects and the exact anti-obesity mechanisms remain unclear. The current limited research provided that Aiye may confer anti-obesity effects through its water-soluble phenolics. However, there was a lack of research that fully elucidated the anti-obesity mechanisms of the exact phenolics in the water extract of Aiye. 

The current research identified the phenolic profile of Aiye and elucidated its anti-obesity mechanisms by combining liquid chromatography-mass spectrometry (LC-MS), network pharmacology, and molecular docking. Network pharmacology has been widely applicable in revealing the therapeutic mechanisms of plant secondary metabolites on various diseases, including allergic rhinitis, inflammatory response, rheumatoid arthritis, etc. [57,58,59]. As for anti-obesity, molecular docking technology is widely applied to evaluate the interaction between compounds and targets by visualizing the intermolecular forces and presenting the binding affinity between compounds and targets [60,61,62]. The water-soluble bioactive components presented higher anti-oxidative capacity than nutraceutical mixtures’ lipid-soluble components [63]. Therefore, in the current research, the phenolics of Aiye were extracted by water. Firstly, phenolics were extracted by water and then identified by LC-MS. Secondly, the identified phenolics were screened by Lipinski rules to ensure their bioavailability and drug-likeness. Thirdly, the targets of phenolics and obesity were collected from different databases, and the protein–protein-interaction (PPI) network of overlapped targets between phenolics and obesity was constructed. The core and central targets were selected for Gene Ontology (GO), Kyoto Encyclopedia of Genes and Genomes (KEGG) enrichment analysis, and molecular docking. GO and KEGG analysis results predicted the anti-obesity mechanisms. The binding affinity and docking details between key phenolics and central targets were achieved through molecular docking. Furthermore, the Aiye-components-targets-obesity-KEGG signaling pathways network was constructed to present the connection between them. The research results predicted the possible anti-obesity mechanisms of Aiye and revealed the possibility of developing anti-obesity drugs from Aiye.

## 2. Materials and Methods

### 2.1. LC-MS Analysis

Qichun County Shenzhou Qiai Biotechnology Co., Ltd. (Qichun, China) provided the Aiye sample, cultivated in Qichun county and harvested during the Dragon Boat Festival in 2022. The fresh Aiye was sun-dried and stored in the warehouse for one year. Based on the traditional Chinese medicine theory, fresh Aiye is unsuitable for medical use. Therefore, after harvest, the sun-dried Aiye was stored for at least one year until further processing and application. A voucher specimen (ID: UIC-FS-2023-03-01) was stored at T8-508 in Food Science Laboratories in BNU-HKBU United International College. Previous studies have verified the anti-obesity effects of Aiye water extract. Therefore, the current research aims to identify the bioactive components and explore the anti-obesity mechanisms from the water extract of Aiye. 

A total of 100.0 g of Aiye was washed twice to remove the dust on it. After soaking in one liter of distilled water for 30 min, it was put into four liters of boiling water for 20 min. After cooling down, the decoction was filtered by cotton gauze. The filtrate was centrifuged under 10,614× *g* for 10 min at 25 °C. After centrifugation, the supernatant passed through the 0.22 μm microfiltration membrane. After that, the rotary evaporation machine (Shanghai Yarong Biochemistry Instrument Factory, RE-52AA, Shanghai, China) concentrated the water to decrease the volume. Finally, the concentrated filtrate was frozen at −80 °C and freeze-dried by the freeze-drying machine (Scientz-18N/A, Ningbo, China). 

The comprehensive chemical profile analysis of the freeze-dried water extract was performed by ultra-performance liquid chromatography–electrospray ionization–tandem mass spectrometry (UPLC-ESI-MS/MS, ExionLC™ AD, https://sciex.com.cn/, accessed on 7 November 2023) [64]. After the first lyophilization, the extract powder was kept in the refrigerator until the LC-MS analysis. Before the LC-MS analysis, the extract powder was lyophilized again to eliminate the moisture caused during storage and preparation. Specifically, the sample was put into a lyophilizer (Scientz-100F, Ningbo, China) for freeze-drying, and then it was ground by a grinder (MM 400, Retsch, Haan, Germany) for 1.5 min at a frequency of 30 Hz. An electronic balance (MS105DM) weighed fifty milligrams of sample powder. Subsequently, 1200 microliters of 70% methanol were mixed with the powder for sample extraction. The 70% methanol solution contained 1 mg/L of internal standard to monitor whether the components were stable during the extraction and whether the instruments were accurate during the detection. The 70% methanol solution with an internal standard was prepared in two steps. Firstly, the internal standard stock solution was prepared by dissolving 5 mg of 2-chlorophenylalanine (J&K Scientific, Beijing, China, CAS: 14091-11-3) in 5 mL of methanol. Secondly, 1 mL of the stock solution was added to 1 L of 70% methanol. The solution was stored at −20 °C before being used. Every 30 min, the mixture was vortexed for 30 s and vortexed six times. After that, the mixture was centrifuged at 16,099× *g* for 3 min. A 0.22 μm microfiltration membrane filtered the supernatant and was ready for UPLC-ESI-MS/MS analysis. 

The parameter setting of the liquid chromatography section was adapted from the method described previously [65]. The column was Agilent SB-C18 (1.8 μm, 2.1 mm × 100 mm). The mobile phase A was 0.1% formic acid in ultrapure water solution, and the mobile phase B was 0.1% formic acid in acetonitrile solution. From 0.00 min to 9 min, the ratio of the mobile phase B was linearly increased from 5% to 95%. After that, the ratio of mobile phase B was kept at 95% until 10 min. From 10 to 11.10 min, the ratio of the mobile phase B decreased from 95% to 5%. The ratio of the mobile phase B was kept as 5% until 14 min. The flow rate of the mobile phase was kept as 0.35 mL/min. The column temperature was set as 40 °C. The injection volume of sample extraction was two micro-liters. 

The parameter setting of the mass spectrometry was adapted from the method described previously [66]. The temperature of ESI was 500 °C. The spray voltage for the positive model was 5500 V, while it was −4500 V for the negative model. The gas I of ionization source, gas II of ionization source, and the curtain gas were 50, 60, and 25 psi, respectively. Multiple reaction monitoring (MRM) was used in the QQQ scanning of metabolites. The collision gas (N_2_) was set as a medium. The de-clustering potential (DP) and collision energy (CE) for each MRM precursor and product ion were optimized. The precursor and product ions were selected to monitor the relevant metabolites at the elution time. 

The mass spectrum data, such as the secondary spectra, retention time, and accurate mass of precursor and product ions of each metabolite, were matched to the database of Metware (Wuhan Metware Biotechnology Co., Ltd., Wuhan, China). The tolerance of the secondary spectrum was set as 20 ppm, and the retention time offset was less than 0.2 min. In this way, the chemical profile of the lyophilized water extract of Aiye was identified.

### 2.2. Drug-Likeness Evaluation

Firstly, the canonical SMILES of the 30 components were obtained from the PubChem database (retrieved from https://pubchem.ncbi.nlm.nih.gov/, accessed on 5 March 2024). Secondly, the canonical SMILES were input into the SwissADME webpage (retrieved from http://www.swissadme.ch/, accessed on 5 March 2024) [67]. From the SwissADME webpage, the molecular weight, the number of hydrogen bond acceptors and donors, the lipophilicity, and the bioavailability of those components were downloaded. Finally, the Lipinski rules were used to screen the drug-likeness properties of those components [68,69] 

### 2.3. Prediction of Protein Targets of Components

The canonical SMILES of each identified component were input into the inquiry box of both the STP (retrieved from http://www.swisstargetprediction.ch/, accessed on 5 March 2024) [26] and the SEA database (retrieved from https://sea.bkslab.org/, accessed on 5 March 2024) [70]. *Homo sapiens* was selected as the species for target prediction. The targets of each component from the two databases were combined, and the repeated targets were removed. The total targets of all the elements were combined for the follow-up analysis. 

### 2.4. Collection of Obesity Targets 

The obesity targets were collected from five databases, which were DrugBank (retrieved from https://go.drugbank.com/, accessed on 31 August 2023) [71], Uniprot (retrieved from https://www.uniprot.org/, accessed on 31 August 2023), OMIM (retrieved from https://www.omim.org/, accessed on 1 September 2023) [72], DisGeNET (retrieved from https://www.disgenet.org/search, accessed on 1 September 2023) [73], and GeneCards (retrieved from https://www.genecards.org/, accessed on 1 September 2023) [74,75]. Only the protein-coding gene targets were selected for the subsequent analysis. 

### 2.5. Targets Overlapping between Components and Obesity

The targets of components and obesity were plotted by the Venny 2.1 online tool (retrieved from https://bioinfogp.cnb.csic.es/tools/venny/, accessed on 5 March 2024) [76]. The overlapped area indicated the potential anti-obesity targets of the identified components. 

### 2.6. Construction of PPI Network 

The multiple anti-obesity targets of components were visualized by STRING 12.0 (retrieved from https://string-db.org/, accessed on 5 March 2024) [77]. *Homo sapiens* was selected for continuing analysis. The database lacked three targets: VEGFA, MDH1, and LDHB. 

For settings, the PPI network was functionally and physically associated. The edge between the two nodes indicated their associations with each other. All the provided sources of interaction between nodes were selected, including experiments, databases, co-expression, etc. The overlapped nodes were separated so that all could be identified. Two formats of the PPI network were exported: tab-separated values (TSVs) and portable network graphic (PNG) formats, respectively. 

### 2.7. Construction of Interaction Networks 

Different interaction networks were constructed by Cytoscape 3.10.1 [78] as follows. 

### 2.8. Construction of PPI Network of Aiye Anti-Obesity Targets for the Screening of Core and Central Targets 

The PPI network containing 483 targets exported from STRING was imported into Cytoscape, and the three independent nodes were excluded: PASK, KLK7, and P2RX3. For the 480 nodes left, the betweenness centrality, closeness centrality, and degree centrality were calculated by CytoNCA [79]. Furthermore, the maximal clique centrality (MCC), maximum neighborhood component (MNC), degree, and closeness were calculated by cytoHubba [80]. 

### 2.9. Construction of Aiye Core Anti-Obesity Targets for Enrichment Analysis 

The nodes whose betweenness centrality, closeness centrality, and degree centrality were all higher than the median were chosen as the core targets, and the interaction network was visualized by Cytoscape [79,81]. Furthermore, the GO and KEGG enrichment analysis of the core targets was performed by Metascape (retrieved from https://metascape.org/gp/index.html#/main/step1, accessed on 5 March 2024) [82]. 

### 2.10. Construction of Aiye Central Targets for Component–Target Docking

The top 10 targets of MCC, MNC, Degree, and Closeness were ranked by cytoHubba, and their overlapping was regarded as the central targets for subsequent docking [81].

### 2.11. GO and KEGG Enrichment Analysis 

The biological processes (BPs), cellular components (CCs), molecular functions (MFs), and KEGG pathway enrichment analysis of core targets were performed on Metascape (retrieved from https://metascape.org/gp/index.html#/main/step1, accessed on 5 March 2024) [82]. *Homo sapiens* were the organisms of enrichment analysis. The minimum overlap was 3, the *p* value cutoff was 0.01, and the minimum enrichment was 1.5. The enrichment analysis results were visualized by bioinformatics (retrieved from https://www.bioinformatics.com.cn/, accessed on 5 March 2024). 

### 2.12. Construction of Aiye-Components-Targets-Obesity-Signaling Pathway Network

The interaction network of the Aiye-components-targets-obesity-signaling pathway was visualized by Cytoscape 3.10.1 [78]. Nodes of different categories were assigned various colors and shapes. The components, targets, and pathways were all plotted in a cycle layout according to the degree centrality. The more central location indicated the higher degree centrality of the node.

### 2.13. Component–Target Docking 

The identified components, whose degree centrality was higher than the average level, were chosen as the docking ligands with receptor targets. The canonical SMILES of those components were converted into the 2-dimensional (2D) structure by ChemDraw (version 22.0.0). The 2D structure of each component was imported into Chem3D (version 22.0.0) to generate the minimized energy form of the compound and output it in PDB format. The PDB format of central targets was downloaded from the Protein Data Bank database (retrieved from https://www.rcsb.org/, accessed on 5 March 2024). The water was removed from targets, and hydrogen was added to both targets and components by Autodock Tools (version 1.5.7) [83,84]. The results were kept as PDBQT format files. A grid box encompassing the target was established, and its binding domain was determined by utilizing AutoDock Vina (version 1.2.5) [85,86]. 

The conformation with the lowest binding affinity was kept for each component–target complex. A binding affinity less than −5 kcal/mol indicated a moderate binding affinity, while a solid binding affinity required an affinity lower than −7 kcal/mol [87]. Pymol and Discovery Studio visualized the docking results with an affinity of less than −7 kcal/mol. The heat map of docking results was plotted by bioinformatics (retrieved from https://www.bioinformatics.com.cn/, accessed on 13 November 2023).

## 3. Results

### 3.1. The Identification of Phenolics through UPLC-ESI-MS/MS

A total of 19.34 g of freeze-dried powder were obtained from the water extract of the 100.0 g dried Aiye sample, resulting in a yield of approximately 19.34%. Before chemical profile analysis, 50 mg of a lyophilized extract was dissolved in 1200 μL of 70% methanol. There were 30 phenolics identified by UPLC-ESI-MS/MS, which belonged to four categories. There were 20 flavonoids, which accounted for 66.67% of the identified phenolics, and 15 of them were flavones, 3 were flavonols, 1 was flavanone, and 1 was isoflavone. Six phenolic acids occupied 20% of the identified phenolics, and three coumarins accounted for 10%. The last one was chromone, which comprised 3.33% of the identified phenolics. The results are presented in Table 1. The secondary mass spectrum of phenolics 1–30 is provided in the Supplementary Materials listed as Figures S1–S30. 

### 3.2. Drug-Likeness Screening of Phenolics 

The molecular weight, number of hydrogen bond acceptor and donor, lipophilicity, bioavailability, and polar surface area of the 30 phenolics were obtained from the webpage of SwissADME (retrieved from http://www.swissadme.ch/, accessed on 5 March 2024) [67,68,69], and 21 of them satisfied the criteria; thus, they were selected for subsequent analysis. The details are represented in Table 2. 

### 3.3. Prediction Targets of Phenolics 

The targets of the 21 phenolics were collected from STP (retrieved from http://www.swisstargetprediction.ch/, accessed on 5 March 2024) [88] and SEA (retrieved from https://sea.bkslab.org/, accessed on 5 March 2024) [70]. There were 611 protein targets identified.

### 3.4. Collection Targets of Obesity 

Eight thousand two hundred twenty-five obesity targets of *Homo sapiens* were identified. Two thousand eight hundred twenty-one obesity targets were collected from DisGeNET [73], and 111 targets were obtained from DrugBank [71]. Furthermore, there were 7506 obesity targets gained from GeneCards [74,75], while 89 targets were acquired from OMIM [72].

### 3.5. The Overlapped Targets between Phenolics and Obesity 

**Figure 1:**
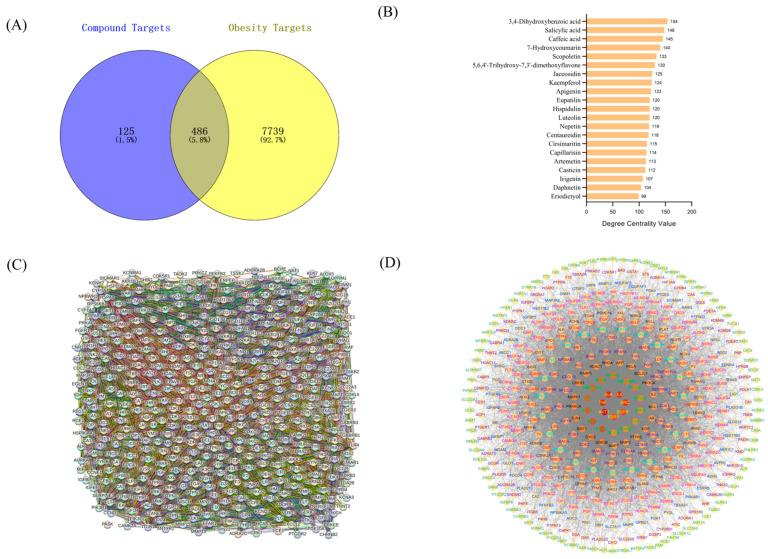
(**A**) The common targets of phenolics and obesity. (**B**) The name and target number of the 21 phenolics. (**C**) The PPI network of the 483 nodes. (**D**) The PPI network formed by the 480 nodes.

The 21 phenolics extracted from Aiye, which satisfied the Lipinski rules, possessed 611 targets. The total number of obesity targets identified was 8225. An analysis using Venny 2.1 software revealed 486 common targets between phenolics and obesity (Figure 1A). The name, chemical structure, number of anti-obesity targets (degree centrality), and pharmacological properties of the 21 phenolics were summarized in Table 3. The degree centrality indicated the edges of a node in a network, and the edges connected different nodes. A phenolic was connected to its anti-obesity targets by the edges. In other words, the degree centrality of a phenolic indicated its number of anti-obesity targets. The phenolics whose target number exceeded the average level were selected for target docking. The name and target number of the 21 phenolics are shown in Figure 1B. 

### 3.6. Construction of PPI Network

The associations among the 486 anti-obesity targets were presented by the STRING 12.0 database (retrieved from https://string-db.org/, accessed on 5 March 2024) [77]. VEGFA, MDH1, and LDHB were the three targets that did not exist in the database. Therefore, they were excluded. The PPI network of the 483 nodes is shown in Figure 1C. PASK, KLK7, and P2RX3 were the three independent nodes without associations with others. For the interacted 480 nodes, the number of edges was 8738. For all the nodes of the PPI network, the average node degree was 36.2, and the average local clustering coefficient was 0.49. The *p*-value of enrichment of the PPI network was less than 1.0 × 10^−16^. The results showed that the nodes of the PPI network were significantly associated.

### 3.7. Construction of Anti-Obesity Targets of Aiye for Core and Central Target Screening

Cytoscape reconstructed the PPI network containing 483 nodes constructed by STRING. The three independent nodes, PASK, KLK7, and P2RX3, were not shown (Figure 1D). The network formed by the 480 nodes contained 8738 edges. The median of betweenness centrality, closeness centrality, and degree centrality were 131.58, 0.447, and 24, respectively. 

Degree centrality indicated the edges of the node, and a higher degree centrality meant more associations with others. Closeness centrality indicated the node’s distance from others; when the closeness centrality of a node was close to 1, it was more centrally located in the network. Betweenness centrality revealed the importance and convenience of a node. A higher betweenness centrality indicated more associations with others. The network containing 480 targets formed 13 cycles, and the internal cycle comprised nodes with higher degree centrality than the external cycle. From outside to inside, the filling color of nodes changed from light yellow to orange red, indicating a continuous increase in the degree centrality of the nodes. The innermost central cycle includes seven protein nodes, of which the degree centrality belongs to the top 7. For every cycle, the color of node labeling was different so that every node could be identified.

### 3.8. Construction of Core Targets for Enrichment Analysis

**Figure 2:**
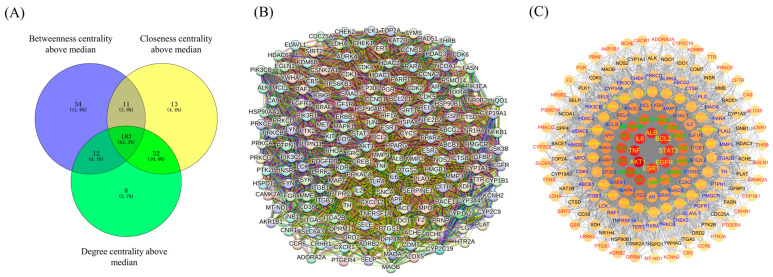
(**A**) Screening of core targets whose betweenness centrality, closeness centrality, and degree centrality were all above the median. (**B**) The PPI network of the 183 core targets constructed by the STRING database. (**C**) The PPI network of the 183 core targets constructed by Cytoscape.

The enrichment analysis was conducted using 183 core targets. The betweenness centrality, closeness centrality, and degree centrality of those core targets were all higher than the median (Figure 2A) [79]. The associations among the 183 nodes were plotted by STRING 12.0 (Figure 2B) [77]. The 183 nodes possessed 4723 edges, and the average node degree was 51.6. The average local clustering coefficient was 0.584. The *p*-value of enrichment of the network was lower than 1.0 × 10^−16^. The data showed that the nodes of the network were significantly associated. 

The network containing 183 nodes was replotted by Cytoscape (Figure 2C). The reconstructed PPI network had 4723 edges and was composed of 7 cycles. From external to internal, the degree centrality of nodes was increased. Meanwhile, for each node, the filling color became deeper and deeper with the increase in its degree centrality. The innermost cycle contained eight protein nodes whose degree centrality belonged to the top 8. For each cycle, the labeling color of nodes was adjusted so that every node could be identified easily. 

### 3.9. Construction of Central Targets of Aiye for Component–Target Docking

**Figure 3:**
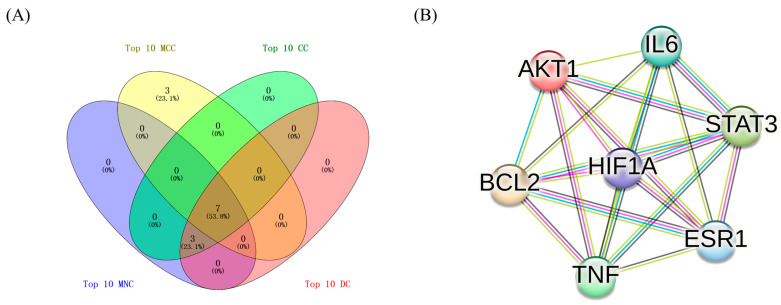
(**A**) Screening of central nodes whose maximal clique centrality (MCC), maximum neighborhood component (MNC), degree and closeness all belonged to the top 10. (**B**) The PPI network of seven central nodes visualized through STRING database.

Seven central nodes (Figure 3A) were selected for molecular docking, whose MCC, MNC, degree, and closeness all belonged to the top 10 [80]. The seven central nodes were plotted by STRING 12.0 [77] (Figure 3B). The seven central nodes possessed 21 edges, and the average node degree was six. The average local clustering coefficient was one, indicating that every node was associated with each other.

### 3.10. GO and KEGG Enrichment Analysis 

Metascape performed the BP, CC, MF, and KEGG pathway enrichment [82]. The 183 core targets were involved in 2287 BP terms, 162 CC terms, 248 MF terms, and 214 KEGG pathways terms. The *p*-value of all the enrichment analysis terms was less than 0.01, and at least three genes were enriched in every term. Furthermore, the gene counts enriched in each term were at least 1.5 times higher than expected by accident. 

**Figure 4:**
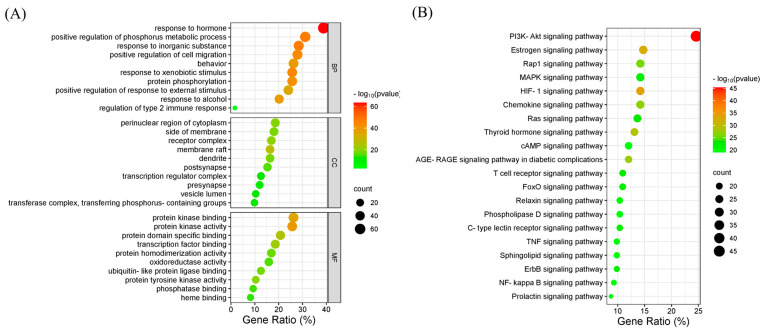
(**A**) Top 10 biological processes (BPs), cellular components (CCs), and molecular functions (MFs). (**B**) The top 20 KEGG signaling pathways.

The top 10 BP, CC, and MF were presented in an enrichment dot bubble (Figure 4A). The top 10 of BP included response to hormones, the positive regulation of the phosphorus metabolic process, response to an inorganic substance, the positive regulation of cell migration, behavior, response to xenobiotic stimulus, protein phosphorylation, the positive regulation of the response to external stimulus, response to alcohol, and the regulation of type 2 immune response. The various biological processes enriched by the core targets indicated phenolics’ broad biological functions. The top 10 of CC included the perinuclear region of cytoplasm, the side of the membrane, receptor complex, membrane raft, dendrite, postsynapse, transcription regulator complex, presynapse, vesicle lumen, and transferase complex, transferring phosphorus-containing groups. Various cellular components revealed that the core targets involved different signaling pathways and played multiple roles in many biological processes. The top 10 of MF were protein kinase binding, protein kinase activity, protein-domain-specific binding, transcription factor binding, protein homodimerization activity, oxidoreductase activity, ubiquitin-like protein ligase binding, protein tyrosine kinase activity, phosphatase binding, and heme binding. The top 10 MF involved protein transcription, phosphorylation, and dephosphorylation.

The top 20 KEGG signaling pathways were shown in the enrichment dot bubble (Figure 4B), which included the PI3K-Akt signaling pathway, estrogen signaling pathway, Rap1 signaling pathway, MAPK signaling pathway, HIF-1 signaling pathway, chemokine signaling pathway, ras signaling pathway, thyroid hormone signaling pathway, cAMP signaling pathway, AGE-RAGE signaling pathway in diabetic complications, and the prolactin signaling pathway, etc. The instability of blood glucose due to the distortion of the MAPK and PI3K/AKT pathway causes diabetes and obesity [110]. The PI3K/AKT pathway imbalance resulted in insulin resistance, followed by obesity and diabetes [111]. The adipogenesis, differentiation, and metabolism were correlated with the prolactin signaling pathway [31]. The activation of the AMPK signaling pathway was triggered by the activation of the cAMP signaling pathway, which was pivotal in the energy balance [112]. The development of obesity and diabetes was correlated with the cAMP and AMPK signaling pathways. The different KEGG signaling pathways indicated that the core targets of phenolics may have multiple effects in regulating signaling cascades related to obesity. The anti-obesity mechanisms of the phenolics extracted from Aiye may have correlated with the BP, CC, MF, and KEGG signaling pathways enriched by the 183 core targets. 

### 3.11. Construction of Aiye-Components-Targets-Obesity-Signaling Pathway Network

**Figure 5:**
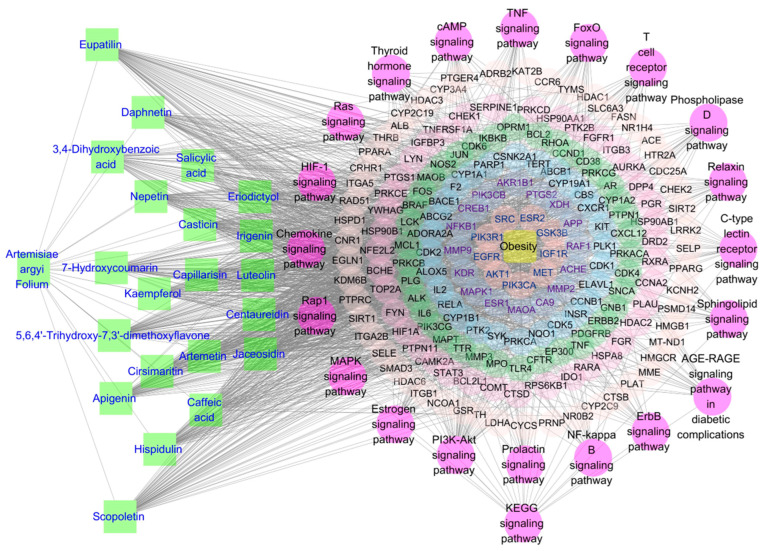
The Aiye-components-targets-obesity-signaling pathway network.

The Aiye-components-targets-obesity-signaling pathway network was constructed by Cytoscape 3.10.1 [78], containing 227 nodes and 1868 edges (Figure 5). Aiye and components were represented by a box filled with green color, and the labeling color was blue. Each of the 21 phenolics formed an edge with the node representing Aiye; meanwhile, they also formed edges with their targets. The signaling pathway was represented by a round node, which was filled with a pink color. The top 20 signaling pathways formed one big cycle surrounding all the core targets and obesity nodes. On the one hand, the 20 signaling pathway nodes formed edges with the node representing the KEGG signaling pathway, and on the other hand, they were all connected with the enriched targets themselves. Signaling pathway nodes surrounded the nodes representing targets and obesity. Nine targets surrounded the obesity node, the innermost cycle of core targets. In total, the 183 core targets formed six cycles. Each cycle had different filling and labeling colors so every node could be identified.

### 3.12. Component–Target Docking 

Autodock Tools (version 1.5.7) [83,84] and AutoDock Vina (version 1.2.5) [85,86] were applied in the docking between the eight phenolics and the seven central targets. The seven central targets were as follows: HIF1A (PDB ID: 4H6J) [113,114], AKT1 (PDB ID: 4GV1) [115], TNF (PDB ID: 2AZ5) [116,117], IL6 (PDB ID: 1ALU) [118], ESR1 (PDB ID: 7UJO) [119], STAT3 (PDB ID: 6NJS) [120], and BCL2 (PDB ID: 4MAN) [121]. 

A lower binding affinity indicated a more stable connection of component–target docking. When the binding affinity was less than −5 kcal/mol, the binding between component and target was moderate. The component–target connection was strong when the binding affinity was less than −7 kcal/mol [87]. Among the 56 docking results, there were 45 whose binding affinity was below −5 kcal/mol. Furthermore, there were 13 docking results whose binding affinity was lower than −7 kcal/mol, including kaempferol-AKT1 complex, kaempferol-BCL2 complex, 5,6,4′-trihydroxy-7,3′-dimethoxyflavone-STAT3 complex, jaceosidin-ESR1 complex, etc. Generally, phenolics showed moderate or strong binding affinity with central targets.

**Figure 6:**
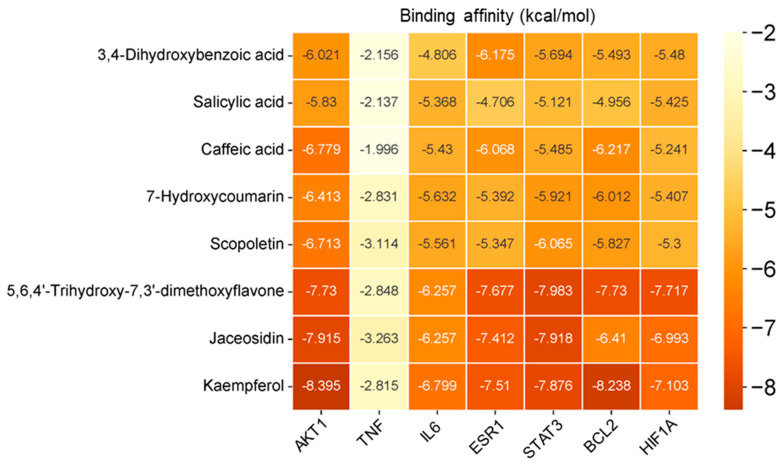
The 56 binding results visualized by a heatmap.

**Figure 7:**
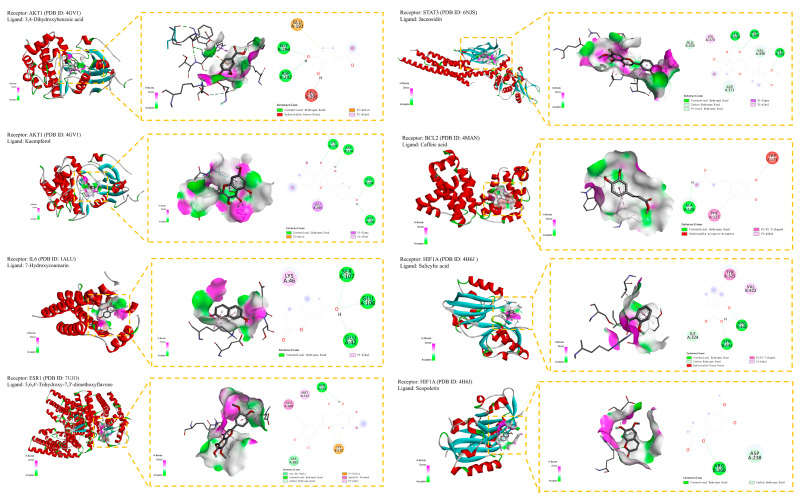
The representative ligand and receptor complex in 2D and 3D form.

The binding results were presented in a heatmap (Figure 6). The Y-axis was the eight phenolics, and the X-axis was the seven targets. The value of binding affinity was shown in the box, and a deeper color of the box indicated a lower binding affinity. The component–target complex whose binding affinity was lower than −5 kcal/mol was presented by Pymol and Discovery Studio. The 2D and 3D results of the representative one were presented (Figure 7). The target HIF1A had the most potent binding with 5,6,4′-trihydroxy-7,3′-dimethoxyflavone, with a binding affinity equal to −7.717 kcal/mol. The binding affinity between BCL2 and kaempferol was −8.238 kcal/mol. STAT3 and 5,6,4′-trihydroxy-7,3′-dimethoxyflavone had a binding affinity equal to −7.983 kcal/mol. The binding affinity between ESR1 and 5,6,4′-trihydroxy-7,3′-dimethoxyflavone was −7.677 kcal/mol. IL6 and kaempferol had a binding affinity equal to −6.799 kcal/mol. AKT1 and kaempferol had a binding affinity equal to −8.395 kcal/mol. The binding affinity between TNF and all the components was beyond −5 kcal/mol, which was not tightly connected.

## 4. Discussion

The current research combined LC-MS, network pharmacology, and molecular docking to explore the possible anti-obesity mechanisms of Aiye. LC-MS was widely applied to determine the bioactive components of fruits, cereals, nuts, and herbal medicine [62,65,66,122,123]. Network pharmacology and molecular docking were usually combined to reveal the molecular mechanisms of disease treatment and elaborate on the compound–target interactions [124,125,126]. In this study, the LC-MS analysis identified thirty phenolics, and 21 of them satisfied the standards of Lipinski’s rules. In total, the 21 phenolics possessed 486 anti-obesity targets. Different phenolics may synergistically participate in weight loss by regulating different targets and signaling pathways. Eight of the 21 phenolics were selected for molecular docking since their anti-obesity targets exceeded the mean value. 

Among the 486 anti-obesity targets, 483 were identified by the String database, so the PPI Network constructed only contained 483 nodes. For the 483 nodes, there were three nodes independent of the others. Therefore, when the PPI network was imported into Cytoscape 3.10.1, those three independent nodes were eliminated, and the updated PPI network contained 480 targets. There were 183 targets whose betweenness centrality, closeness centrality, and degree centrality were above the median. Those targets were the core targets for GO and KEGG enrichment analysis. Seven targets, including MCC, MNC, degree, and closeness, ranked in the top ten, which indicated that the seven targets were centrally located in the PPI network with the most extensive interactions and connections with other targets. Thus, the seven targets were selected as central targets for molecular docking. 

The enrichment analysis includes the BP, CC, MF, and KEGG analyses. The top 10 of BP include response to hormones, positive regulation of the phosphorus metabolic process, and protein phosphorylation. Gut hormones involved in the insulinotropic and anorexigenic effects could be potential anti-obesity targets [127]. The targets of phenolics are enriched in response to hormones, which may indicate their potential weight control effects. 

The top 10 of CC showed that the 183 core targets were significantly enriched in different cellular components, including the perinuclear region of cytoplasm, side of membrane, and membrane raft. Various cellular components revealed that phenolics may trigger multiple biological processes at different cellular sites. The top 10 of MF involve the binding and activity of protein kinase, transcription factor binding, and phosphatase binding. Protein kinase results in the phosphorylation of proteins, which influences subsequent signaling pathways [128]. By targeting core targets, phenolics may affect the transcription, post-translation modifications, phosphorylation, and dephosphorylation of proteins. Therefore, the anti-obesity mechanism of phenolics may regulate the transcription and modifications of obesity-relevant proteins. 

The top 20 KEGG signaling pathways include the PI3K-Akt signaling pathway, estrogen signaling pathway, MAPK signaling pathway, cAMP signaling pathway, and prolactin signaling pathway. The abnormality of the MAPK and PI3K/AKT signaling pathways caused the fluctuation of blood glucose, which finally developed into diabetes and obesity [110]. Insulin resistance, obesity, and diabetes are caused by the imbalance of the PI3K/AKT pathway [111]. The prolactin signaling pathway regulates lipogenesis, differentiation, and metabolism [31]. Energy balance is regulated by the AMPK and cAMP signaling pathways, and the two pathways are correlated with the development of obesity and diabetes [112]. The top KEGG signaling pathway enriched by the core targets of phenolics is tightly related to obesity. By targeting the core targets, the phenolics of Aiye are promising in combating obesity. 

For 56 component–target complexes, there were 45 docking with a binding affinity of less than −5 kcal/mol. Furthermore, there were 13 docking results whose binding affinity was lower than −7 kcal/mol, including the kaempferol-AKT1 complex, kaempferol-BCL2 complex, 5,6,4′-trihydroxy-7,3′-dimethoxyflavone-STAT3 complex, jaceosidin-ESR1 complex, etc. Generally, phenolics showed moderate or strong binding affinity with central targets. Network pharmacology and molecular docking have theoretically elucidated the anti-obesity mechanisms of Aiye. The next step is to perform in vitro and in vivo studies to verify the anti-obesity effects of the identified phenolics. 

## 5. Conclusions

The current research identified 30 phenolics from Aiye by LC-MS. Furthermore, the anti-obesity targets and signaling pathways were predicted by combining network pharmacology and molecular docking. In total, 486 anti-obesity targets were found, and after screening, 183 core targets and seven central targets were selected. The 183 core targets performed GO and KEGG enrichment analysis, while the seven central targets went through molecular docking with the eight phenolics whose degree centrality was above the average level. Research outcomes indicated that the core targets played various roles in different biological processes, such as responding to hormone and protein phosphorylation, which may contribute to the anti-obesity effects. The MF enriched by the 183 core targets involved protein transcription, phosphorylation, and dephosphorylation. The core targets of phenolics were enriched in the PI3K-Akt signaling pathway, MAPK signaling pathway, prolactin signaling pathway, and cAMP signaling pathway, all correlated with obesity. The outcomes of molecular docking showed that the binding affinity between the eight phenolics and the seven central targets was, overall, moderate or strong. The research outcomes theoretically proved the feasibility of Aiye’s phenolics in treating obesity. The in vitro and in vivo experiments should be designed to verify the anti-obesity effects and mechanisms of the identified phenolics. This may bring light to develop anti-obesity drugs with fewer side-effects compared with the current anti-obesity medications.

## Figures and Tables

**Table 1:** The 30 phenolics in Aiye identified by LC-MS.

No.	Retention Time (min)	Compounds	CAS ID	Collision Energy (eV)	Classification	Precursor ion (Da)	Product ion (Da)	Peak Area
1	2.20	1-Caffeoylquinic acid	1241-87-8	−20	Phenolic acids	353.09	191.06	69,797,865.10
2	2.30	7-Hydroxycoumarin	93-35-6	30	Coumarins	163.04	89.04	1,763,845.40
3	2.50	3,4-Dihydroxybenzoic acid	99-50-3	−18	Phenolic acids	153.02	109.03	1,662,701.81
4	3.10	Salicylic acid	69-72-7	−30	Phenolic acids	137.03	108.02	21,278,543.18
5	3.30	Schaftoside	51938-32-0	47	Flavones	565.16	529.13	7,564,970.69
6	3.40	Caffeic acid	331-39-5	−20	Phenolic acids	179.03	135.05	12,700,935.26
7	3.50	Isoschaftoside	52012-29-0	30	Flavones	565.16	409.09	11,713,194.36
8	3.50	Daphnetin	486-35-1	30	Coumarins	179.03	133.03	4,308,737.58
9	3.80	Vitexin	3681-93-4	−30	Flavones	431.10	311.05	4,787,487.98
10	3.80	Rutin	153-18-4	30	Flavonols	611.16	303.05	917,616.01
11	4.00	Cynarin	30964-13-7	30	Phenolic acids	517.13	163.04	102,685,412.20
12	4.00	Isorhoifolin	552-57-8	30	Flavones	579.17	271.06	10,644,918.47
13	4.00	Isoquercitrin	482-35-9	−30	Flavonols	463.09	300.03	617,610.61
14	4.10	Scopoletin	92-61-5	−30	Coumarins	191.04	176.01	5,348,582.99
15	4.20	Isochlorogenic acid B	14534-61-3	−30	Phenolic acids	515.12	353.09	15,763,996.82
16	5.00	Luteolin	491-70-3	−40	Flavones	285.04	151.00	59,730,853.76
17	5.20	Eriodictyol	552-58-9	−20	Flavanones	287.05	135.04	20,620,576.66
18	5.20	Nepetin	520-11-6	30	Flavones	317.07	302.04	6,988,200.11
19	5.60	5,6,4′-Trihydroxy-7,3′-dimethoxyflavone	-	30	Flavones	331.08	298.04	8,576,062.16
20	5.70	Apigenin	520-36-5	30	Flavones	271.06	153.02	2,783,945.85
21	5.70	Kaempferol	520-18-3	−30	Flavonols	285.04	151.00	539,429.30
22	5.80	Hispidulin	1447-88-7	30	Flavones	301.07	286.05	3,710,550.04
23	5.80	Capillarisin	56365-38-9	30	Chromone	317.07	302.04	1,337,073.68
24	5.90	Jaceosidin	18085-97-7	30	Flavones	331.08	316.06	16,825,514.25
25	5.90	Centaureidin	17313-52-9	30	Flavones	361.09	303.05	3,058,589.54
26	6.00	Irigenin	548-76-5	40	Isoflavones	361.09	310.00	461,645.02
27	6.40	Cirsimaritin	6601-62-3	20	Flavones	315.09	254.06	5,762,441.22
28	6.70	Eupatilin	22368-21-4	30	Flavones	345.10	330.07	35,641,022.22
29	6.90	Casticin	479-91-4	30	Flavones	375.11	299.06	28,620,099.27
30	7.60	Artemetin	479-90-3	30	Flavones	389.12	331.08	8,618,699.94

Collision energy; negative value means negative ionization mode.

**Table 2:** The physicochemical properties, lipophilicity, and drug-likeness of the 30 phenolics.

No.	Compounds	Lipinski Rules				Lipinski’s Violations	Bioavailability Score	TPSA (Å^2^)
		MW	HBA	HBD	MLogP			
		<500	<10	≤5	≤4.15	≤1	> 0.1	<140
1	1-Caffeoylquinic acid	354.31	9	*6*	−1.05	1	0.11	*164.75*
2	7-Hydroxycoumarin	162.14	3	1	1.04	0	0.55	50.44
3	3,4-Dihydroxybenzoic acid	154.12	4	3	0.40	0	0.56	77.76
4	Salicylic acid	138.12	3	2	0.99	0	0.85	57.53
5	Schaftoside	*564.49*	*14*	*10*	−3.97	*3*	0.17	*250.97*
6	Caffeic acid	180.16	4	3	0.70	0	0.56	77.76
7	Isoschaftoside	*564.49*	*14*	*10*	−3.97	*3*	0.17	*250.97*
8	Daphnetin	178.14	4	2	0.45	0	0.55	70.67
9	Vitexin	432.38	*10*	*7*	−2.02	*2*	0.55	*181.05*
10	Rutin	*610.52*	*16*	*10*	−3.89	*3*	0.17	*269.43*
11	Cynarin	*516.45*	*12*	*7*	−0.35	*3*	0.11	*211.28*
12	Isorhoifolin	*578.52*	*14*	*8*	−2.96	*3*	0.17	*228.97*
13	Isoquercitrin	464.38	*12*	*8*	−2.59	*2*	0.17	*210.51*
14	Scopoletin	192.17	4	1	0.76	0	0.55	59.67
15	Isochlorogenic acid B	*516.45*	*12*	*7*	−0.35	*3*	0.11	*211.28*
16	Luteolin	286.24	6	4	−0.03	0	0.55	111.13
17	Eriodictyol	288.25	6	4	0.16	0	0.55	107.22
18	Nepetin	316.26	7	4	−0.31	0	0.55	120.36
19	5,6,4’-Trihydroxy-7,3’-dimethoxyflavone	330.29	7	3	−0.07	0	0.55	109.36
20	Apigenin	270.24	5	3	0.52	0	0.55	90.90
21	Kaempferol	286.24	6	4	−0.03	0	0.55	111.13
22	Hispidulin	300.26	6	3	0.22	0	0.55	100.13
23	Capillarisin	316.26	7	3	0.37	0	0.55	109.36
24	Jaceosidin	330.29	7	3	−0.07	0	0.55	109.36
25	Centaureidin	360.31	8	3	−0.35	0	0.55	118.59
26	Irigenin	360.31	8	3	−0.35	0	0.55	118.59
27	Cirsimaritin	314.29	6	2	0.47	0	0.55	89.13
28	Eupatilin	344.32	7	2	0.17	0	0.55	98.36
29	Casticin	374.34	8	2	−0.12	0	0.55	107.59
30	Artemetin	388.37	8	1	0.11	0	0.55	96.59

MW, molecular weight (g/mol); HBA, hydrogen bond acceptor; HBD, hydrogen bond donor; LogP, lipophilicity; Bioavailability score, the ability of a drug or other substance to be absorbed and used by the body; TPSA, topological polar surface area. To pass the screening, a compound should simultaneously satisfy the requirements of Lipinski’s violations (≤1), bioavailability score (>0.1), and TPSA (Å^2^) (<140). The italic values with strikethrough disobeyed the standards.

**Table 3:** The name, chemical structure, number of anti-obesity targets (degree centrality), and pharmacological properties of the 21 phenolics.

Name	Chemical Structure	Anti-Obesity Targets	Pharmacological Properties	References
7-Hydroxycoumarin	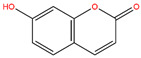	140	Hepatoprotection, nephroprotection, antioxidation, and anti-inflammation effects	[32,33]
3,4-Dihydroxybenzoic acid	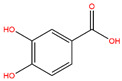	154	Cellular protection effects combating ultraviolet B radiation, nematicidal activity	[89,90]
Salicylic acid	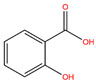	148	Hepatoprotection and nephroprotection effects	[91,92]
Caffeic acid	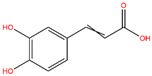	145	Skeletal muscle atrophy alleviation, memory dysfunction attenuation	[93,94]
Daphnetin	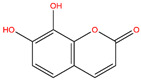	104	Pulmonary inflammation and fibrosis alleviation, airway inflammation amelioration	[34,35,95]
Scopoletin	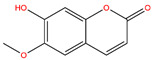	133	Hepatoprotection effects, glucotoxicity alleviation	[53,54,96]
Luteolin	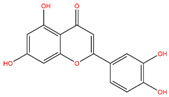	120	Endometritis alleviation, antitumor effects	[22,23,97]
Eriodictyol	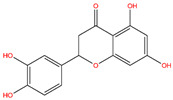	99	Nephroprotection effects, antitumor effects	[24,98]
Nepetin	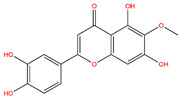	119	Atopic dermatitis attenuation, airway inflammation amelioration, anti-allergic property	[36,37,38]
5,6,4′-Trihydroxy-7,3′-dimethoxyflavone	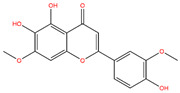	130	Anti-inflammation and anti-oxidation effects	[39]
Apigenin	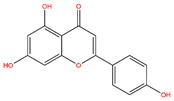	123	Anti-cancer, anti-inflammation, insulin resistance amelioration	[25,40,52]
Kaempferol	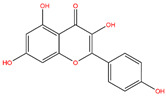	124	Anti-oxidation, anti-inflammation, exercise performance improvement, physical fatigue alleviation	[41,42,43]
Hispidulin	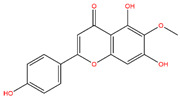	120	Anti-inflammation, anti-microbial effects	[44,45,46,99]
Capillarisin	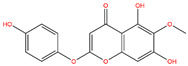	114	Anti-asthmatic activity, neuro-protection effects	[100,101]
Jaceosidin	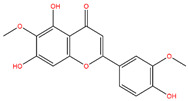	125	Anti-inflammation and hepatic fibrosis modulation, anti-tumor	[19,20,21,47]
Centaureidin	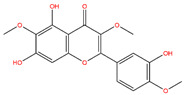	118	Immunomodulatory effects, vasorelaxant activity	[102,103]
Irigenin	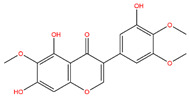	107	Lung injury amelioration, anti-inflammation, anti-cancer effects	[48,104]
Cirsimaritin	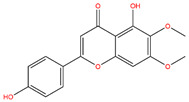	115	Colitis alleviation, anti-diabetic activities, anti-inflammation, anti-oxidation, and hepatoprotection effects	[49,50,51]
Eupatilin	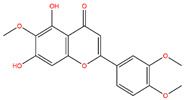	120	Anti-tumor effects, anti-osteoporosis, gastrointestinal hemorrhage prevention, hyperuricemia attenuation, nephroprotection	[26,105,106,107]
Casticin	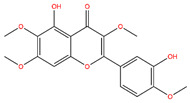	112	Anti-osteoporosis effects, cartilage damage amelioration, anti-tumor activities	[27,108,109]
Artemetin	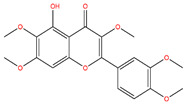	113	Anti-cancer effects	[28,29]

## Data Availability

The data presented in this study are available on request from the corresponding author.

## Supplementary Materials

The following supporting information can be downloaded at: https://www.mdpi.com/article/10.3390/life14060656/s1, Figures S1–S30: The secondary mass spectrum of phenolics 1–30.

## Abbreviations

Aiye	*Artemisiae argyi* Folium
BP	Biological processes
BMI	Body mass index
CC	Cellular components
CDC	Center for Disease Control and Prevention
CE	Collision energy
DP	De-clustering potential
ESI	Electrospray ionization
GO	Gene ontology
KEGG	Kyoto encyclopedia of genes and genomes
LC-MS	Liquid chromatography-mass spectrometry
MCC	Maximal Clique Centrality
MNC	Maximum Neighborhood Component
MF	Molecular functions
MRM	Multiple reaction monitoring
PPI	Protein–protein-interaction
SEA	Similarity ensemble approach
STP	SwissTargetPrediction
MS/MS	Tandem mass spectrometry
UPLC	Ultra performance liquid chromatography
2D	2-Dimensional
