# Supplementary figures and images for: Elucidation of Anti-Obesity Mechanisms of Phenolics in Artemisiae argyi Folium (Aiye) by Integrating LC-MS, Network Pharmacology, and Molecular Docking
Source: Life (Basel). 2024 May 22;14(6):656. doi: 10.3390/life14060656 (PMC11205026; doi:10.3390/life14060656)

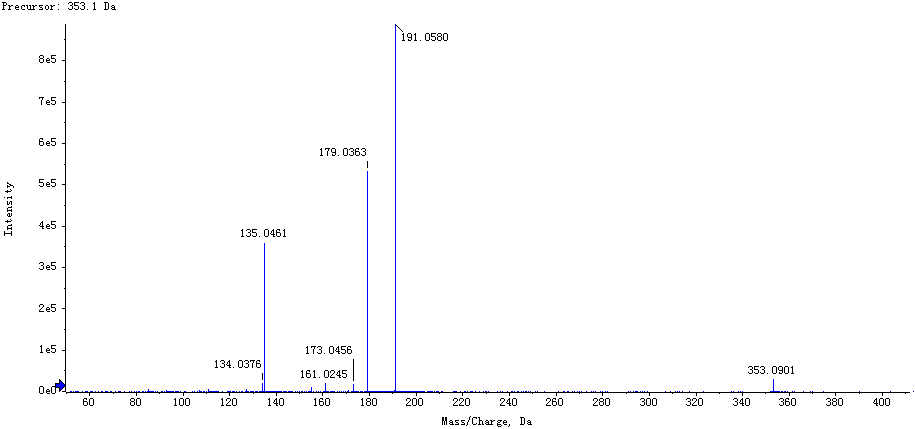

Supplement: Supplementary file 1 [file life-14-00656-s001.zip › The secondary mass spectrum of phenolics 1-30 is provided in the supplementary material listed as S1-S30/S1.png]

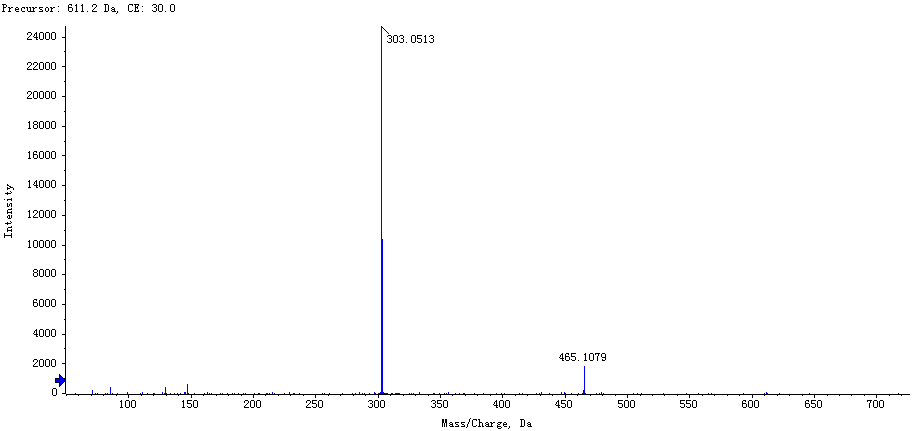

Supplement: Supplementary file 1 [file life-14-00656-s001.zip › The secondary mass spectrum of phenolics 1-30 is provided in the supplementary material listed as S1-S30/S10.png]

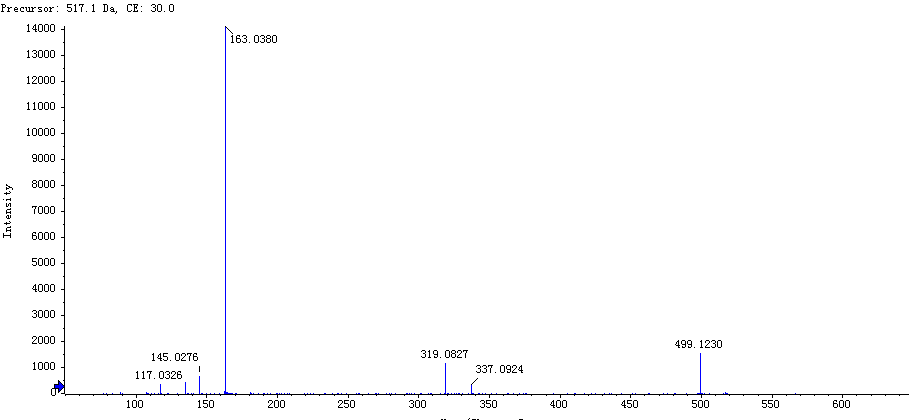

Supplement: Supplementary file 1 [file life-14-00656-s001.zip › The secondary mass spectrum of phenolics 1-30 is provided in the supplementary material listed as S1-S30/S11.png]

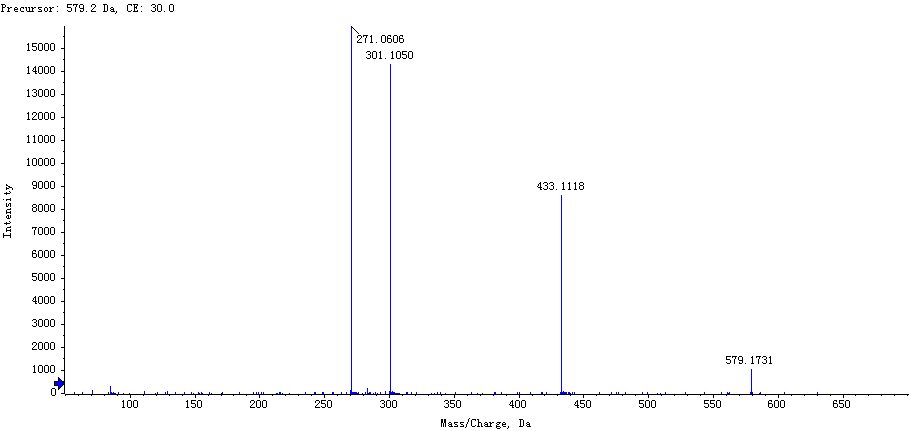

Supplement: Supplementary file 1 [file life-14-00656-s001.zip › The secondary mass spectrum of phenolics 1-30 is provided in the supplementary material listed as S1-S30/S12.png]

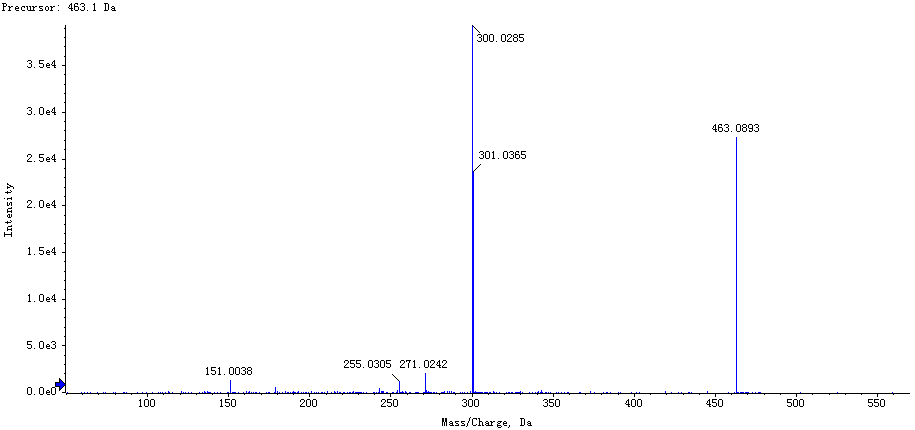

Supplement: Supplementary file 1 [file life-14-00656-s001.zip › The secondary mass spectrum of phenolics 1-30 is provided in the supplementary material listed as S1-S30/S13.png]

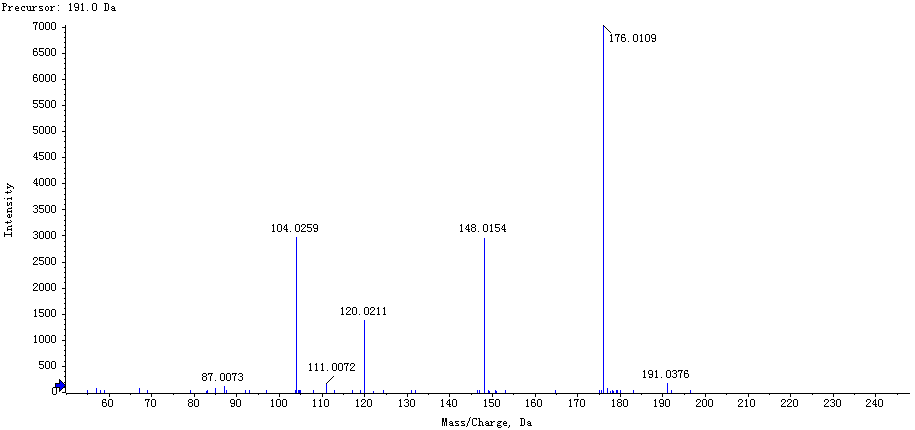

Supplement: Supplementary file 1 [file life-14-00656-s001.zip › The secondary mass spectrum of phenolics 1-30 is provided in the supplementary material listed as S1-S30/S14.png]

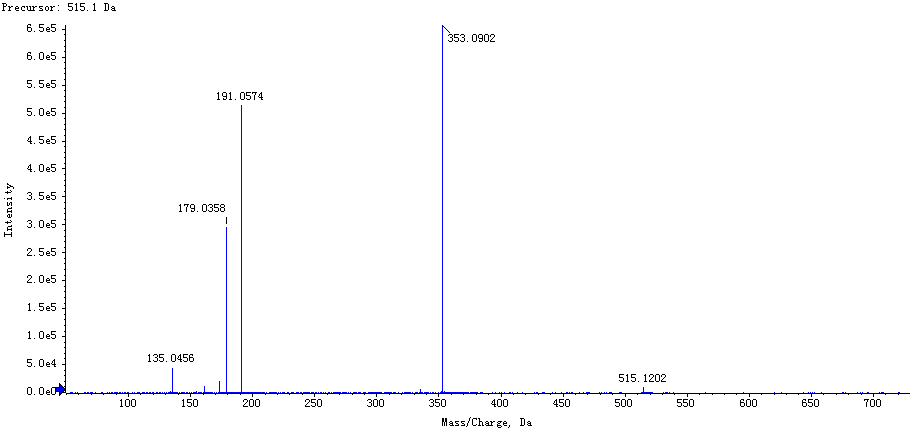

Supplement: Supplementary file 1 [file life-14-00656-s001.zip › The secondary mass spectrum of phenolics 1-30 is provided in the supplementary material listed as S1-S30/S15.png]

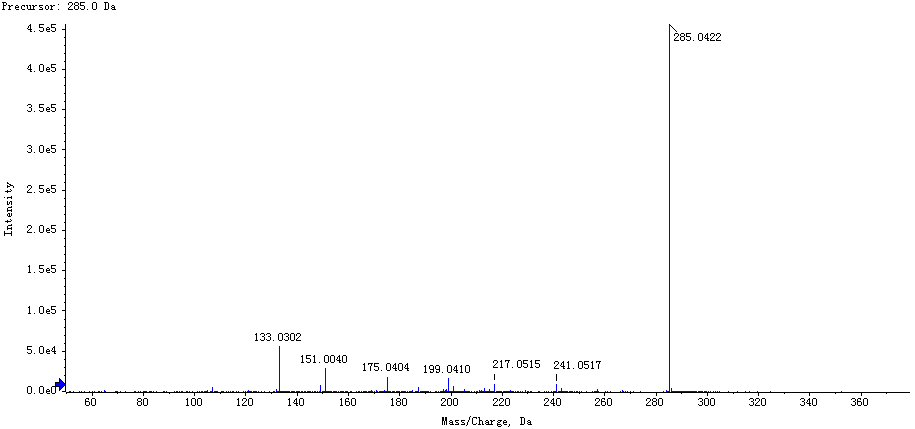

Supplement: Supplementary file 1 [file life-14-00656-s001.zip › The secondary mass spectrum of phenolics 1-30 is provided in the supplementary material listed as S1-S30/S16.png]

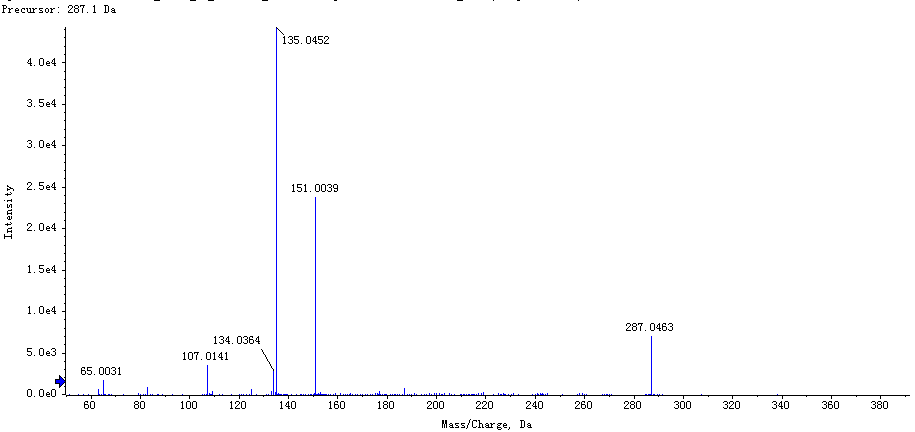

Supplement: Supplementary file 1 [file life-14-00656-s001.zip › The secondary mass spectrum of phenolics 1-30 is provided in the supplementary material listed as S1-S30/S17.png]

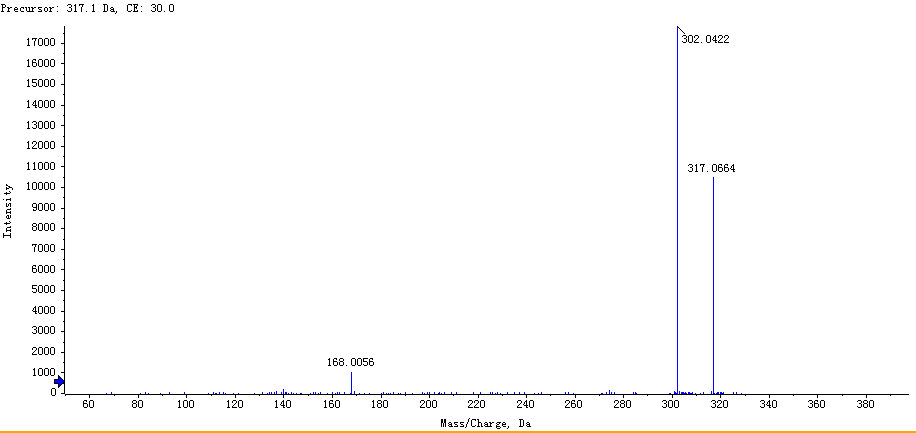

Supplement: Supplementary file 1 [file life-14-00656-s001.zip › The secondary mass spectrum of phenolics 1-30 is provided in the supplementary material listed as S1-S30/S18.png]

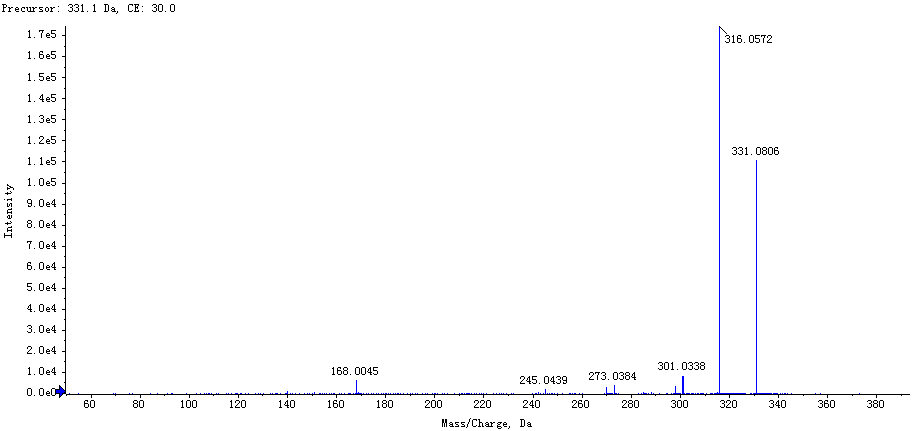

Supplement: Supplementary file 1 [file life-14-00656-s001.zip › The secondary mass spectrum of phenolics 1-30 is provided in the supplementary material listed as S1-S30/S19.png]

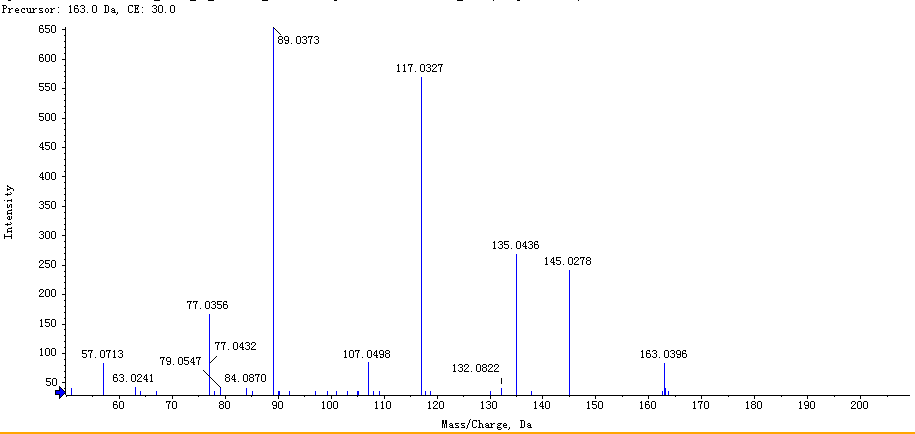

Supplement: Supplementary file 1 [file life-14-00656-s001.zip › The secondary mass spectrum of phenolics 1-30 is provided in the supplementary material listed as S1-S30/S2.png]

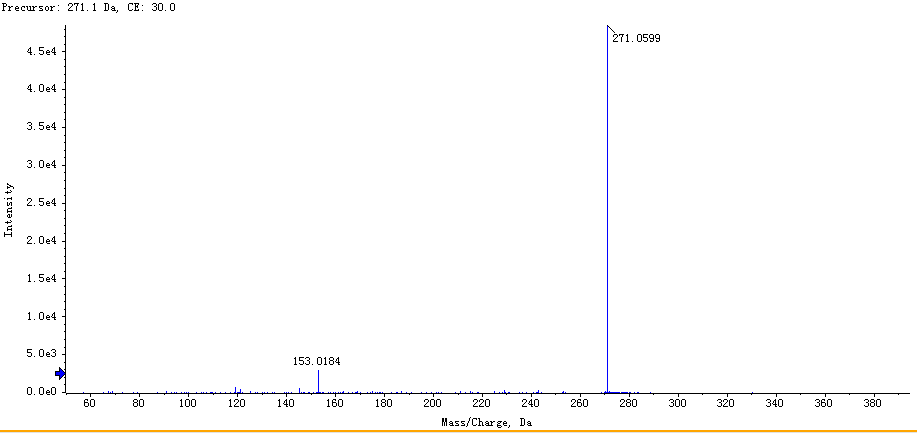

Supplement: Supplementary file 1 [file life-14-00656-s001.zip › The secondary mass spectrum of phenolics 1-30 is provided in the supplementary material listed as S1-S30/S20.png]

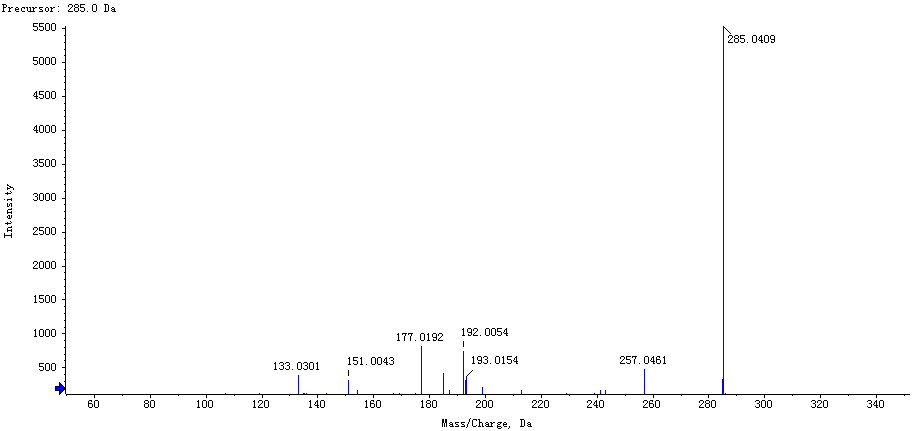

Supplement: Supplementary file 1 [file life-14-00656-s001.zip › The secondary mass spectrum of phenolics 1-30 is provided in the supplementary material listed as S1-S30/S21.png]

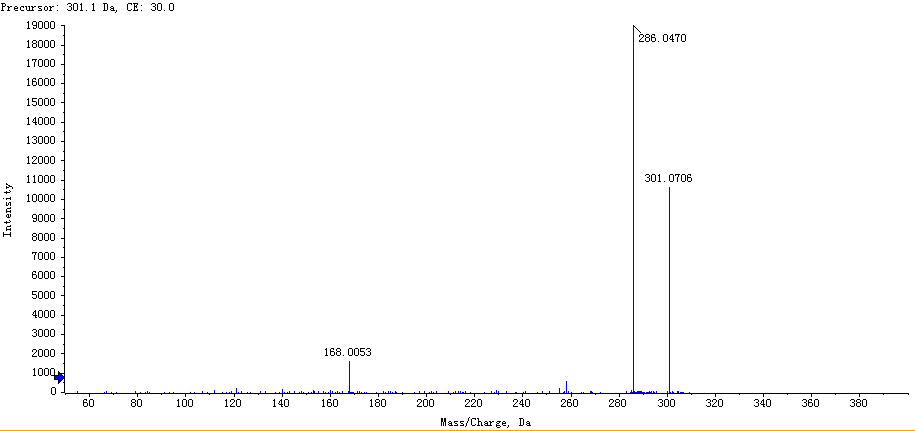

Supplement: Supplementary file 1 [file life-14-00656-s001.zip › The secondary mass spectrum of phenolics 1-30 is provided in the supplementary material listed as S1-S30/S22.png]

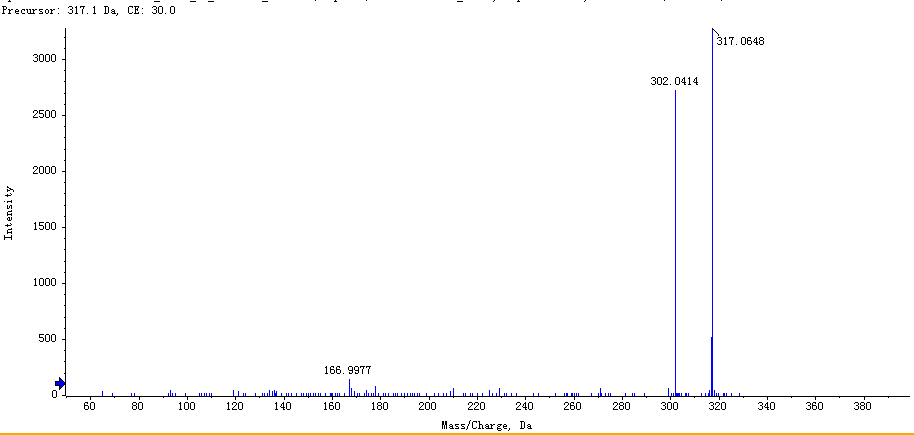

Supplement: Supplementary file 1 [file life-14-00656-s001.zip › The secondary mass spectrum of phenolics 1-30 is provided in the supplementary material listed as S1-S30/S23.png]

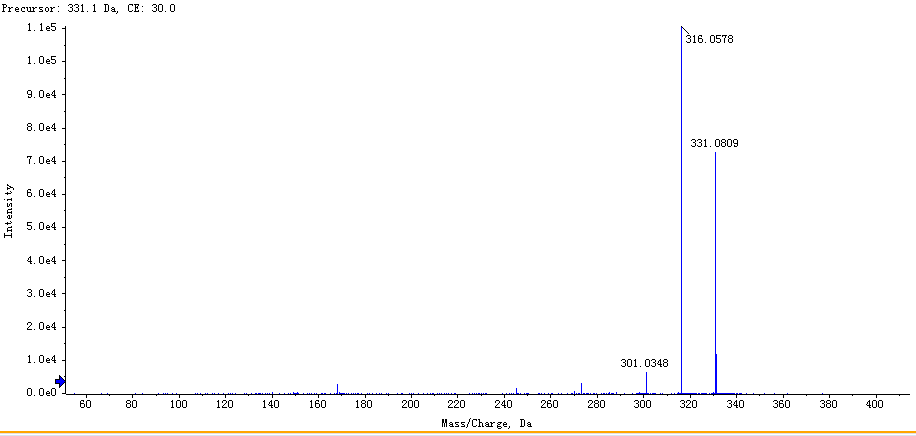

Supplement: Supplementary file 1 [file life-14-00656-s001.zip › The secondary mass spectrum of phenolics 1-30 is provided in the supplementary material listed as S1-S30/S24.png]

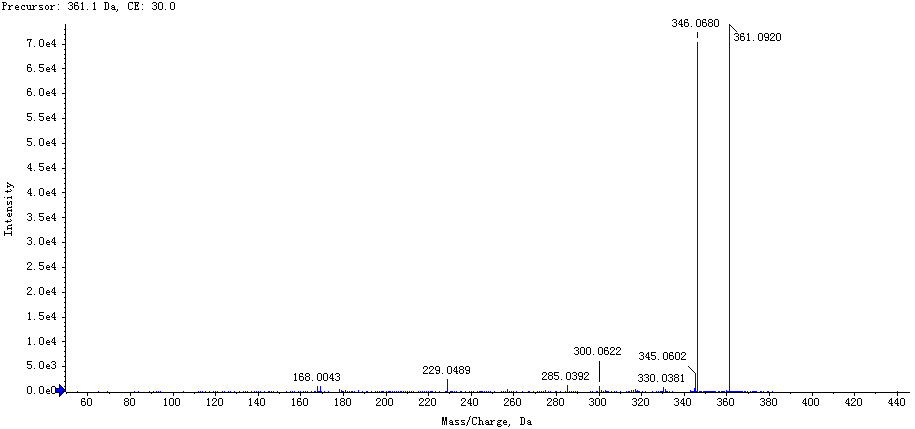

Supplement: Supplementary file 1 [file life-14-00656-s001.zip › The secondary mass spectrum of phenolics 1-30 is provided in the supplementary material listed as S1-S30/S25.png]

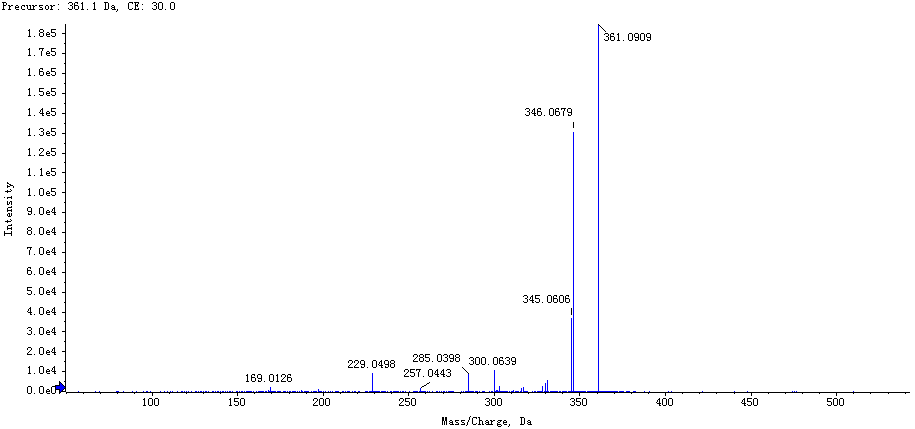

Supplement: Supplementary file 1 [file life-14-00656-s001.zip › The secondary mass spectrum of phenolics 1-30 is provided in the supplementary material listed as S1-S30/S26.png]

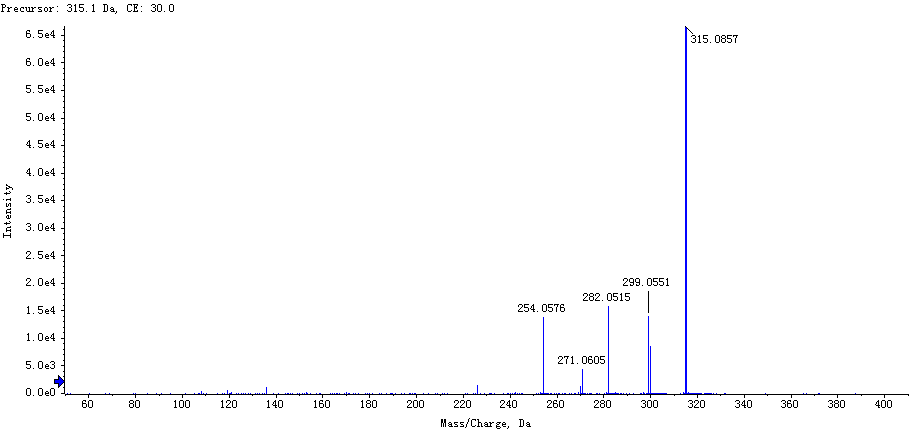

Supplement: Supplementary file 1 [file life-14-00656-s001.zip › The secondary mass spectrum of phenolics 1-30 is provided in the supplementary material listed as S1-S30/S27.png]

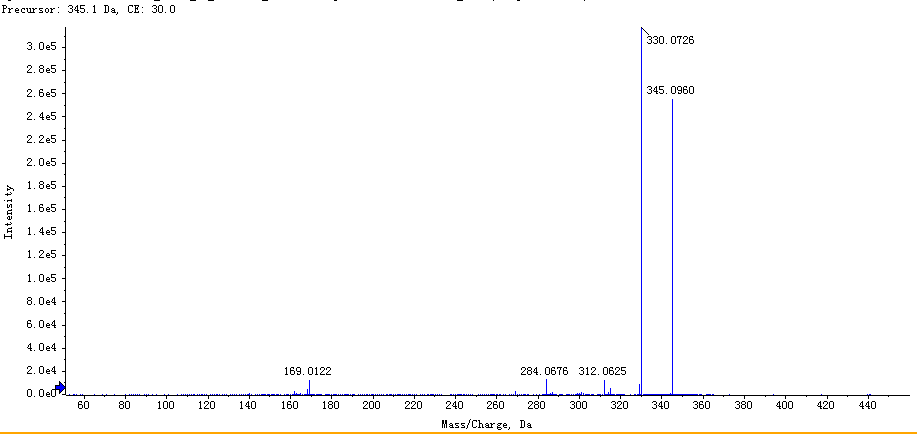

Supplement: Supplementary file 1 [file life-14-00656-s001.zip › The secondary mass spectrum of phenolics 1-30 is provided in the supplementary material listed as S1-S30/S28.png]

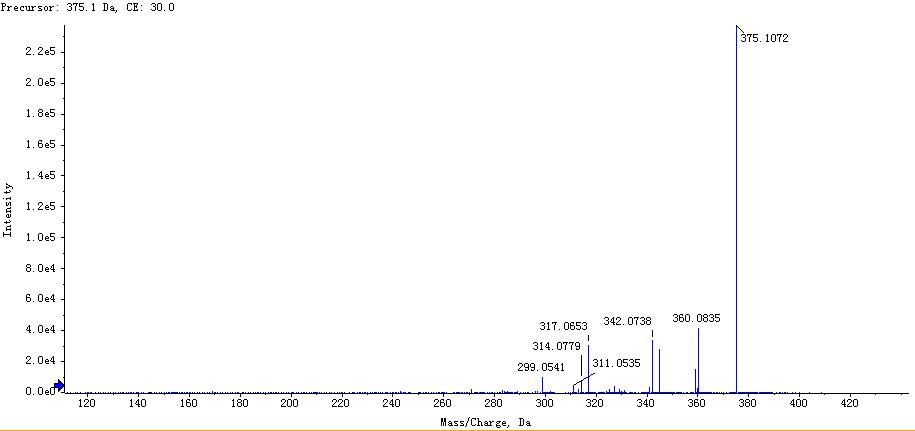

Supplement: Supplementary file 1 [file life-14-00656-s001.zip › The secondary mass spectrum of phenolics 1-30 is provided in the supplementary material listed as S1-S30/S29.png]

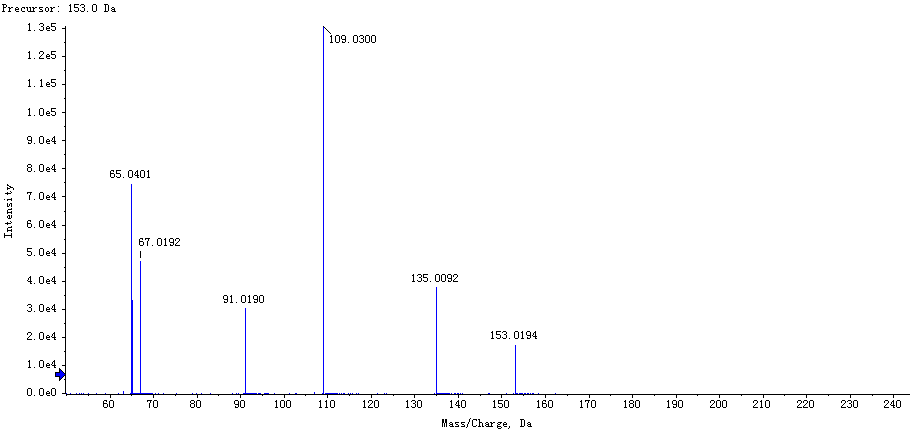

Supplement: Supplementary file 1 [file life-14-00656-s001.zip › The secondary mass spectrum of phenolics 1-30 is provided in the supplementary material listed as S1-S30/S3.png]

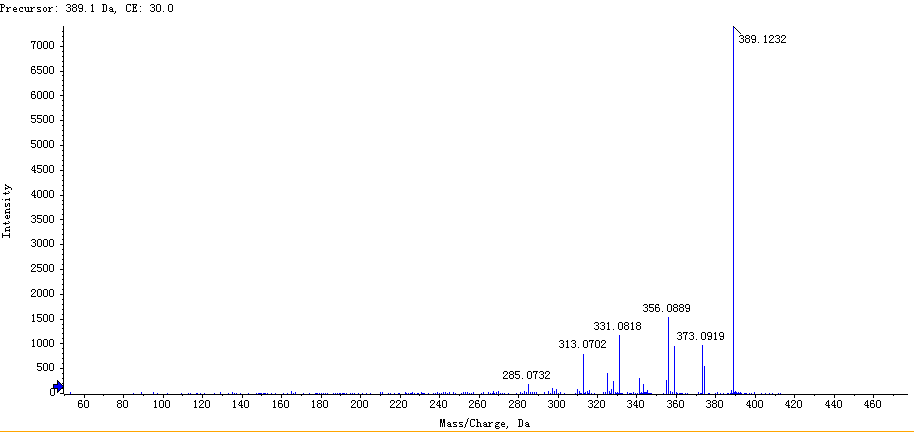

Supplement: Supplementary file 1 [file life-14-00656-s001.zip › The secondary mass spectrum of phenolics 1-30 is provided in the supplementary material listed as S1-S30/S30.png]

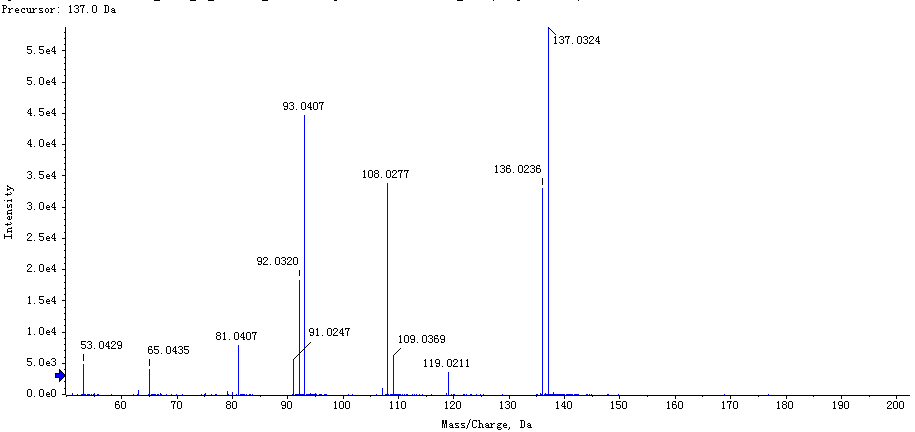

Supplement: Supplementary file 1 [file life-14-00656-s001.zip › The secondary mass spectrum of phenolics 1-30 is provided in the supplementary material listed as S1-S30/S4.png]

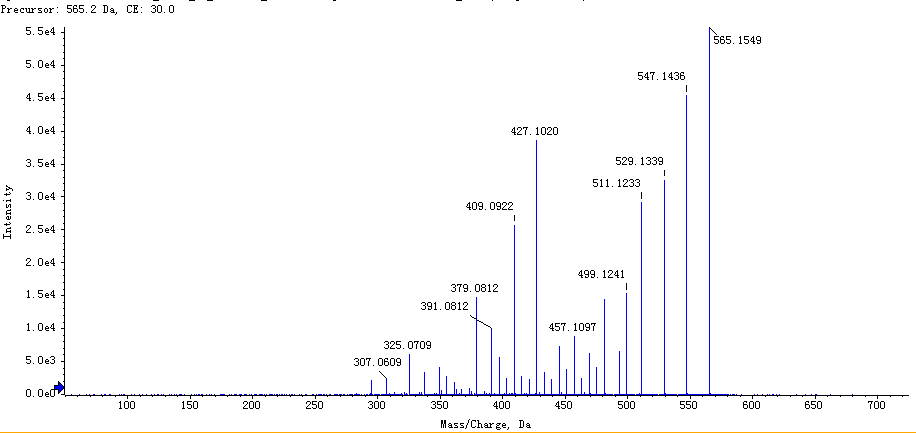

Supplement: Supplementary file 1 [file life-14-00656-s001.zip › The secondary mass spectrum of phenolics 1-30 is provided in the supplementary material listed as S1-S30/S5.png]

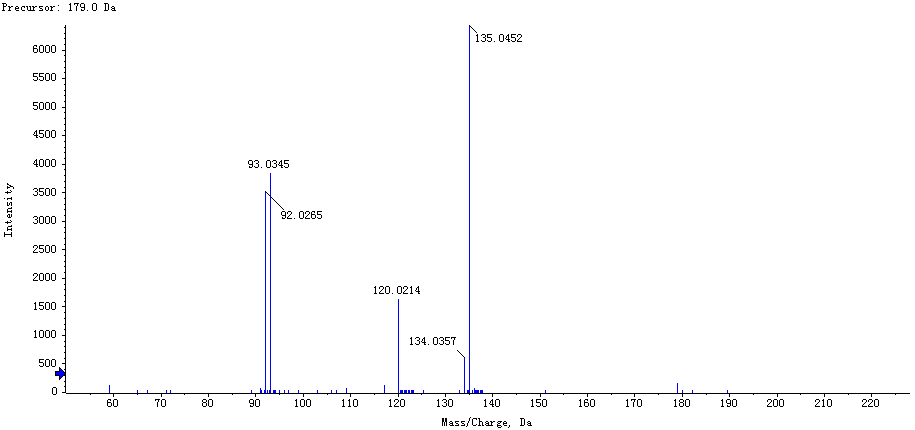

Supplement: Supplementary file 1 [file life-14-00656-s001.zip › The secondary mass spectrum of phenolics 1-30 is provided in the supplementary material listed as S1-S30/S6.png]

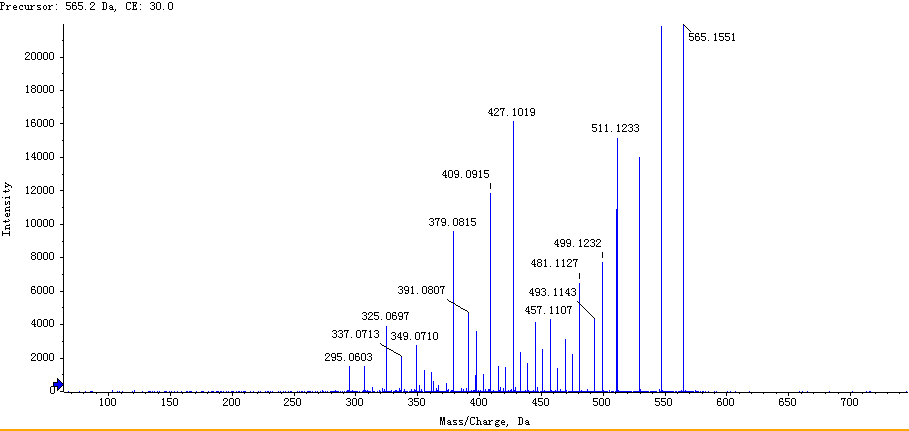

Supplement: Supplementary file 1 [file life-14-00656-s001.zip › The secondary mass spectrum of phenolics 1-30 is provided in the supplementary material listed as S1-S30/S7.png]

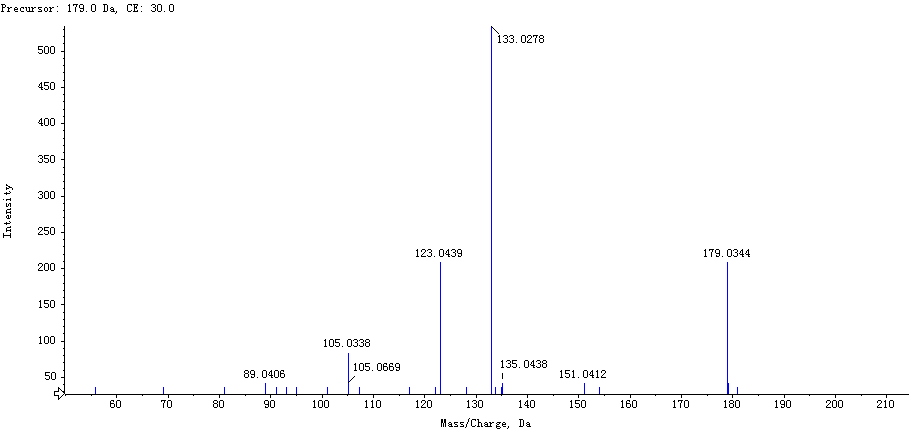

Supplement: Supplementary file 1 [file life-14-00656-s001.zip › The secondary mass spectrum of phenolics 1-30 is provided in the supplementary material listed as S1-S30/S8.png]

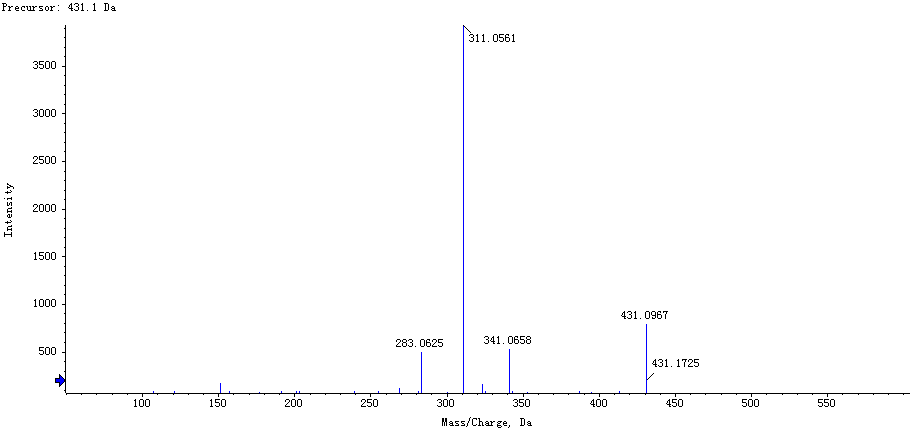

Supplement: Supplementary file 1 [file life-14-00656-s001.zip › The secondary mass spectrum of phenolics 1-30 is provided in the supplementary material listed as S1-S30/S9.png]
